# What makes cancer stem cell markers different?

**DOI:** 10.1186/2193-1801-2-301

**Published:** 2013-07-04

**Authors:** Uwe Karsten, Steffen Goletz

**Affiliations:** Glycotope GmbH, Robert-Rössle-Str.10, D-13125 Berlin-Buch, Germany

**Keywords:** Stem cells, Cancer stem cells, Glycosylation, Thomsen-Friedenreich antigen, Therapeutic targets

## Abstract

Since the cancer stem cell concept has been widely accepted, several strategies have been proposed to attack cancer stem cells (CSC). Accordingly, stem cell markers are now preferred therapeutic targets. However, the problem of tumor specificity has not disappeared but shifted to another question: how can cancer stem cells be distinguished from normal stem cells, or more specifically, how do CSC markers differ from normal stem cell markers? A hypothesis is proposed which might help to solve this problem in at least a subgroup of stem cell markers. Glycosylation may provide the key.

## Background

The cancer stem cell hypothesis (Reya et al. 2001; Al-Hajj et al. 2003; Dalerba et al. 2007; Lobo et al. 2007) proposes that tumors - analogous to normal tissues (Blanpain and Fuchs 2006) - grow and develop from a distinct subpopulation of cells named “cancer stem cells” or “cancer-initiating cells”. Stem cells are able to manage, by asymmetric cell division, two conflicting tasks, self-renewal on the one hand, and (restricted) proliferation and differentiation on the other hand. *Cancer stem cells (CSC)* are thought to be transformed stem or progenitor cells with novel properties such as enhanced proliferation, enhanced mobility and limited ability for differentiation.

Cancer stem cells differ considerably from the majority of cells of the tumor mass. It is assumed that the unlimited growth capacity of the tumor as well as the capability to develop metastases rest on the CSC population. Cancer stem cells divide relatively slowly and are essentially drug-resistant, two properties which make them refractory to conventional chemotherapy. The acceptance of the CSC concept therefore demands re-evaluation and potentially re-direction of cancer therapies: instead of trying solely to reduce the tumor mass, the CSC subset should be specifically targeted. This aim implies the need to search for CSC-specific therapeutic target marker molecules. Cancer stem cells are, however, in many aspects very similar to normal stem cells. They apparently express the same markers as normal stem cells. Therapies aimed at cancer stem cells therefore have a new problem: how to target cancer stem cells and leave normal stem cells intact? Or, in other words, how can CSC markers be distinguished from markers of normal stem cells?

### Stem cell markers

In recent years considerable effort has been invested in the detection and characterization of stem cell markers. The result is that there are now an overwhelming and steadily increasing number of such marker molecules. Some markers are indeed more or less specific for different types of stem cells, for example, markers that differentiate embryonic from adult stem cells or pluripotent from progenitor cells. With the exception of pluripotent embryonic stem cells all other stem cells carry, in addition, lineage-specific markers. Stem cells are also defined by the absence of certain markers. Contemplating these data, several questions arise. First, as already mentioned, almost all markers of normal stem cells are also found on cancer stem cells. Examples are shown in Table 1. This, of course, poses a problem with respect to their potential use as therapeutic targets. Ectopic (non-lineage) expression of stem cell markers on cancer cells does not resolve the therapeutic dilemma. Currently the best option for a therapeutic target would be to rely on onco-fetal stem cell markers which are not expressed on normal adult stem cells. Otherwise there is at present no clear-cut distinction available between normal and cancer stem cell markers. Even at the level of regulatory miRNA clusters, identical patterns were observed (Shimono et al. 2009). Several stem cell markers are upregulated in cancer, e.g. ABCG or Bmi-1. In other instances, mutations have been detected (Lobo et al. 2007; Guo et al. 2008). In some cases isotypes of stem cell markers are preferentially expressed on tumor cells (e.g. CD44v, Günthert et al. 1991; or ALDH1A3, Marcato et al. Marcato et al. 2011), although this issue is not finally settled (Zöller 2011). We believe that a different, more general approach should be considered.

**Table 1:** **Examples of non-carbohydrate stem cell markers which are also cancer stem cell markers**

Marker	Description	Expressed on		Selected references
	***Cellular localization***	Normal stem or progenitor cells	Cancer stem cells	
ALDH1	Aldehyde dehydrogenase *Cytoplasma*	AdSC (breast)	CSC (breast and other carcinomas	1
Bmi-1	Polycomb protein *Cytoplasma*	HSC, NSC, AdSC (intestine, breast, prostate)	CSC (breast, prostate cancer, neuroblastomas, leukemias)	2, 3
CD29	Integrin-β1 *Membrane*	AdSC (breast)	CSC (breast, colon cancer)	4, 5
CD34	Adhesion protein *Membrane*	HSC, MSC, HProgC, EnProgC	CSC (leukemias, sarcomas)	6-11
CD44	Hyaluronan receptor, adhesion protein *Membrane*	HSC, HProgC, PSC	CSC (many carcinomas)	12-16
CD90	Thy-1 *Membrane*	ProgC (thymus), MSC	CSC (breast cancer, glioblastomas)	17, 18
CD117	SCF receptor *Membrane*	ProgC	CSC (breast, ovarian, lung cancer, glioblastomas)	16, 19
CD133	Prominin-1 *Membrane*	HSC, NSC, AdSC (colon)	CSC (many carcinomas, glioblastomas, melanomas)	20-24
CDw338	ABCG2, Bcrp1 ABC transporter, permitting multi-drug resistance *Membrane*	ESC, HSC, AdSC	CSC (breast, lung cancer, glioblastomas, melanomas)	25, 26
Nestin	Class VI intermediate filament protein *Cytoplasma*	NSC, ProgC (brain), HProgC	CSC (glioblastomas, melanomas)	27, 28
Oct-4	Transcription factor *Cytoplasma*	ESC, iPSC	CSC (many carcinomas)	29, 30

This table lists only a few examples (exclusively human data) and selected references. It is not intended as a full review.

Abbreviations: *AdSC* adult stem cell, *CSC* cancer stem cell, *EnProgC* endothelial progenitor cell, *ESC* embryonic stem cell, *HProgC* hematopoietic progenitor cell, *HSC* hematopoietic stem cell, *ProgC* progenitor cell, *PSC* pluripotent stem cell, *iPSC* induced pluripotent stem cell.

,Ginestier et al. 2007; 2, Sangiorgi and Capecchi Sangiorgi and Capecchi 2008; 3, Lukacs et al. Lukacs et al. 2010; 4, Pontier and Muller Pontier and Muller 2009; 5, Taddei et al. Taddei et al. 2008; 6, Krause et al. Krause et al. 1996; 7, Furness et al. Furness et al. 2006; 8, Tardio Tardio 2009; 9, Annaloro et al. Annaloro et al. 2011; 10, Srour et al. Srour et al. 1991; 11, Basso and Timeus Basso and Timeus 1998; Günthert et al. 12, Günthert et al. Günthert et al. 1991; Zöller 13, Zöller Zöller 2011; 14, Singh et al. Singh et al. 2001; 15, Takaishi et al. Takaishi et al. 2009; 16, Zhang et al. Zhang et al. 2008; 17, Augello et al. Augello et al. 2010; 18, Salcido et al. Salcido et al. 2010; 19, Ponnusamy and Batra Ponnusamy and Batra 2008; 20, Liu et al. Liu et al. 2006; 21, Mizrak et al. Mizrak et al. 2008; 22, Ricci-Vitani et al. Ricci-Vitani et al. 2007; 23, Kemper et al. Kemper et al. 2010; O’Brien et al. 24, O’Brien et al. O’Brien et al. 2007; 25, Bunting Bunting 2002; 26, Monzani et al. Monzani et al. 2007; 27, Krupkova et al. Krupkova et al. 2010; Dell’Albani 28, Dell’Albani Dell’Albani 2008; 29, Monk and Holding Monk and Holding 2001; 30, Carpenter et al. Carpenter et al. 2003.

### Hypothesis: what makes CSC markers different?

Most stem cell markers described so far are proteins. A relatively small number of stem cell markers have been shown to be glycans bound to proteins or lipids (Table 2). Glycans are known to be developmentally regulated (Solter and Knowles 1978; Muramatsu 1988; Fenderson and Andrews 1992; Cao et al. 2001), and are often altered on tumor cells (Hakomori 1989; Cao et al. 1995; Dabelsteen 1996; Cao et al. 1997; Brockhausen 1999; Le Pendu et al. 2001; Cao et al. 2008). The question arises whether glycans may be able to play a role as stem cell markers in a more comprehensive sense. Interestingly, the glycosylation of stem cell markers has so far not been systematically examined.

**Table 2:** **Carbohydrate stem cell markers**

Marker	Description	Expression on stem cell-like populations	References
H type 1	SSEA-5, stage-specific embryonic antigen-5; carried on proteins Fucα1-2Galβ1-3GlcNAcβ1-	PSC, iPSC; CSC (germ cell carcinomas)	1
CD15	Lewis X, SSEA-1, stage-specific embryonic antigen-1; carried on lipids or proteins Galβ1-4[Fucα1-3]GlcNAcβ1-3Galβ1-	ESC, NSC, MSC; CSC (globlastomas)	2-7
CD60a	GD3; ganglioside NeuAcα2-8NeuAcα2-3Galβ1-4Glcβ1-	NSC; CSC (differentiated germ cell carcinomas, melanomas)	7, 8
CD77	Gb3, P^k^ antigen, Burkitt lymphoma antigen (BLA); globoside Galα1-4Galβ1-4Glcβ1-	CSC (Burkitt lymphoma, breast cancer, germ cell carcinomas)	8, 9
CD173	H type 2; carried on proteins or lipids Fucα1-2Galβ1-4GlcNAcβ1-	ESC cell lines, HProgC, MSC	9-11, 13
CD174	Lewis Y; carried on proteins or lipids Fucα1-2Galβ1-4[Fucα1-3]GlcNAcβ1-	HProgC; CSC (breast cancer)	9, 11
CD175	Tn antigen; carried on proteins GalNAcα1-	ESC cell lines; onfFN	12,13
CD176	TF, Thomsen-Friedenreich antigen, core-1; carried on proteins Galβ1-3GalNAcα1-	ESC; CSC (diverse carcinomas and leukemias); onfFN	12-14
GD2	OFA-I-2; ganglioside GalNAcβ1-4[NeuAcα2-8NeuAcα2-3]Galβ1-4Glcβ1-	NSC, MSC; CSC (differentiated germ cell carcinomas, breast cancer, melanomas)	7, 8, 10, 15
Gb4	Globoside GalNAcβ1-3Galα1-4Galβ1-4Glcβ1-	CSC (germ cell carcinomas)	8
Gb5	SSEA-3, stage-specific embryonic antigen-3; globoside Carries TFβ (the β-anomer of TF) Galβ1-3GalNAcβ1-3Galα1-4Galβ1-4Glcβ1-	ESC, MSC, iPSC; CSC (breast cancer, germ cell carcinomas)	4, 8, 16-19
Sialyl-Gb5	SSEA-4, stage-specific embryonic antigen-4, GL7; globoside NeuAcα2-3Galβ1-3GalNAcβ1-3Galα1-4Galβ1-4Glcβ1-	ESC, MSC, iPSC, ProgC (breast); CSC (germ cell carcinomas)	4, 8, 16, 17, 19-21
Globo-H	Carried on proteins or lipids Fucα1-2Galβ1-3GalNAcβ1-3Galα1-4Gal-	CSC (breast cancer)	18
TRA-1-60	Tumor-recognition antigen; carried on protein Sialylated keratan sulfate proteoglycan	ESC, MSC; CSC (teratocarcinomas)	4, 19, 22

Abbreviations: *CSC* cancer stem cell, *ESC* embryonic stem cell, *HProgC* hematogenic progenitor cell, *iPSC* induced pluripotent stem cell, *MSC* mesenchymal stem cell, *NSC* neuronal stem cell, *onfFN* oncofetal fibronectin, *ProgC* progenitor cell, *PSC* pluripotent stem cell.

,Tang et al. 2011; 2, Solter and Knowles Solter and Knowles 1978; 3, Son et al. Son et al. 2009; 4, Huang et al. Huang et al. 2009; 5, Hennen and Faissner Hennen and Faissner 2012; 6, Riethdorf et al. Riethdorf et al. 2006; 7, Yanagisawa et al. Yanagisawa et al. 2011; 8, Wenk et al. Wenk et al. 1994; 9, Cao et al. Cao et al. 2001; 10, Lin et al. Lin et al. 2010b; Schäfer et al. 11, Schäfer et al. Schäfer et al. 2011; 12, Matsuura et al. Matsuura et al. 1988; 13, Wearne et al. Wearne et al. 2008Lin et al. 2010a; 15, Battula et al. Battula et al. 2012; 16, Kannagi et al. Kannagi et al. 1983; 17, Henderson et al. Henderson et al. 2002; 18, Chang et al. Chang et al. 2008; 19, Carpenter et al. Carpenter et al. 2003; 20, Gang et al. Gang et al. 2007; 21, LaBarge et al. LaBarge et al. 2007; 22, Badcock et al. Badcock et al. 1999.

For many years we have been interested in the Thomsen-Friedenreich antigen or, more precisely, epitope (TF; CD176), which is an onco-fetal glycan structure (Galβ1-3GalNAcα1-). Although known since the mid-twenties of the last century, it was only in 1975 that Georg F. Springer discovered that this otherwise common cryptic structure is exposed (unmasked) on tumor cells (Springer et al. 1975; Springer 1984). We and others have developed monoclonal antibodies towards TF (Clausen et al. 1988; Karsten et al. 1995; Goletz et al. 2003) and examined its expression on different types of tumor tissues (Itzkowitz et al. 1989; Langkilde et al. 1992; Cao et al. 1995; Cao et al. 1999; Cao et al. 2000; Baldus et al. 2000; Goletz et al. 2003; Cao et al. 2008) as compared to their corresponding normal tissues (Cao et al. 1996). As a result of comprehensive studies it can be stated that in adults TF is a tumor marker of exceptional specificity. Among normal tissues, TF is expressed on activated T cells (Hernandez et al. 2007).

TF does not exist as a separate entity, but as part of a larger carbohydrate structure (*O*-glycan core-1) carried by many glycoproteins primarily of the mucin-type. In the case of tumor cells, these glycans are truncated or otherwise modified, and the core-1 structure (Galβ1-3GalNAcα1-) becomes exposed. Knowing that the glycosylation machinery of tumor cells is generally disturbed (Brockhausen 1999), one might expect that TF is expressed on most if not all glycoproteins of a tumor cell. However, this is not the case. During recent years several carrier molecules have been identified, and it was found that TF is in fact expressed on a very restricted number of proteins of a given tumor type (in most cases one or very few: Matsuura et al. 1988; Zebda et al. 1994; Singh et al. 2001; Baba et al. 2007; Cao et al. 2008). An even greater surprise to us was the fact that almost all TF carrier proteins identified so far turned up as known stem cell markers (Table 3). There are very few exceptions to this statement. The most remarkable exception is oncofetal fibronectin (onfFN, Matsuura et al. 1988), which is characterized by a single *O*-glycosylation (either TF or Tn) at a specific sequence. OnfFN is not a CSC marker *per se*, but an indicator and promoter of epithelial-mesenchymal transition (EMT) of epithelial cancer cells to secondary stem cell-like cells (Ding et al. 2012). A second example are two TF carrying glycoproteins (140 and 110 kDa) found in melanoma cells strongly correlated with high metastatic activity (Zebda et al. 1994). It is not known but conceivable that these proteins are in fact stem cell markers.

**Table 3:** **Carrier molecules of the Thomsen-Friedenreich antigen (TF, CD176)**

Marker	General description/expression on normal stem cells	Expression on cancer stem cells	TF-carrying CSC marker (source)
CD34	Transmembrane protein 105-120 kDa Adhesion protein Immature hematopoietic stem/progenitor cells, endothelial progenitor cells^2^	Leukemias, sarcomas^2^	AML cell line KG-1 (1)
CD44	Hyaluronan receptor, H-CAM, epican, phagocytic glycoprotein-1 80-95 kDa Adhesion protein, binds hyaluronic acid Hematopoietic and non-hematopoietic stem/progenitor cells^2^	Cancer of colon, breast, ovary, lung, liver, stomach, etc.^2^	Colon cancer cell line HT29 (2), lung, breast, and liver cancer (3) Carries also Lewis Y (4)
CD45	Leucocyte common antigen (LCA) 180-240 kDa Hematopoietic stem cells (7)	Glioblastomas (5)	Acute T cell leukemia cell line Jurkat (6)
CD164	MGC-24, endolyn 80 kDa Mucin-like glycoprotein Hematopoietic progenitor cells (10)	Gastric and prostate cancer (8, 9)	Gastric cancer cell line KATO-III (8)
CD227	Mucin-1, MUC1, EMA, PEM >200 kDa Heavily glycosylated mucin MUC1-C interacts with regulatory pathways Hematopoietic progenitor cells (13)	Breast cancer (MCF7) side population (12), gastric cancer, AML (13), multiple myelomas (14)	Breast cancer (11); gastric cancer cell line KATO-III (8)
MAGP^1^	Membrane glycoproteins from human melanoma cell lines 140 kDa (MAGP1), 110 kDa (MAGP2)	Highly metastatic melanoma cell lines (15)	Highly metastatic cell lines (e.g. T1C3) derived from M4Be (15)

Abbreviations: *AML* acute myelogenous leukemia, *CSC* cancer stem cell, *MUC1-C* cytoplasmic domain of MUC1.

^1^Not identical with microfibril-associated glycoproteins, also abbreviated MAGP (16).

^2^References see Table 1.

,Cao et al. 2008; 2, Singh et al. Singh et al. 2001; 3, Lin et al. Lin et al. 2010a; 4, Lin et al. Lin et al. 2010b; 5, Kang and Kang Kang and Kang 2007; 6, Baba et al. Baba et al. 2007; 7, Poppema et al. Poppema et al. 1996; 8, Masuzawa et al. Masuzawa et al. 1992; 9, Havens et al. Havens et al. 2006; 10, Watt et al. Watt et al. 2000; 11, Lloyd et al. Lloyd et al. 1996; 12, Engelmann et al. Engelmann et al. 2008; 13, Fatrai et al. Fatrai et al. 2008; 14, Cloosen et al. Cloosen et al. 2006; 15, Zebda et al. Zebda et al. 1994; 16, Gibson et al. Gibson et al. 1999.

These data and other more general considerations led us to propose the following hypothesis.

1. During the process of malignant transformation from a normal stem or progenitor cell to a cancer stem cell, stem cell glycoprotein markers undergo alterations in their glycosylation.
2. As a consequence, cancer stem cells carry cancer-specific glycans.
3. This appears to be a selective process. Accordingly, these cancer-specific glycans are CSC makers.
4. Changes in stem cell marker glycosylation contribute to the altered biological behavior of these cells.

In brief, we propose that cancer stem cell markers differ from their normal counterparts by the expression of tumor-specific glycans.

In order to substantiate the suggestion that CD176 (Thomsen-Friedenreich antigen) is specifically carried on CSC markers, we have recently performed a study on lung, breast and liver cancer cell lines as well as on tissue sections, in which we examined the co-expression of CD176 with the stem cell markers CD44 and CD133 (Lin et al. 2010a). In tissue sections of all three cancer types 5–30% of cells revealed co-expression of CD176/TF with CD44. Corresponding cell lines confirmed these data but showed greater variability in the number of co-expressing cells. This is not surprising since cell lines *in vitro*, and especially cancer cell lines, are the subject of manifold variation, selection and evolution processes. More importantly, we were able to provide direct evidence by a sandwich ELISA that CD44 is indeed the carrier molecule for CD176/TF in lung, breast and liver cancer cells (Lin et al. 2010a), confirming earlier data from colorectal carcinoma (Singh et al. 2001).

Other data support the proposed hypothesis or are at least not at odds with it.

The cancer stem cell concept implies that metastatic spread is, in principle, restricted to CSCs. In fact, metastases show in most cases a higher percentage of TF-positive cells or of TF-positive cases (Cao et al. 1995). Disseminated breast cancer cells in the bone marrow (DTC-BM, identified as cytokeratin^+^/MUC1^+^) are in almost all cases (96%) positive for CD176/TF (Schindlbeck et al. 2005). This is remarkable, since sections of primary tumors often show a mosaic of TF-positive and TF-negative cells (which is to be expected if TF is a CSC marker). In the light of our hypothesis the expression of TF on DTC might be interpreted as indicating that these cells are cancer stem cells, and thereby able to generate distant metastases. With respect to claim #4 of our hypothesis, it is interesting to note that a number of studies demonstrate the involvement of CD176/TF in metastasis formation (Beuth et al. 1988; Okuno et al. 1993; Shigeoka et al. 1999; Cao et al. 1995). Several modes of TF-mediated adhesion mechanisms leading to metastasis have been described. One is the binding of CD176/TF carrying cells to asialoglycoprotein receptors (ASGPR) in the liver (Schlepper-Schäfer and Springer 1989), which is confirmed by clinical (Cao et al. 1995) and experimental data (Shigeoka et al. 1999). Another TF-mediated mechanism, which leads to hematogenic metastatic spread, has also been described (Yu et al. 2007), and could be experimentally inhibited with TF-carrying anti-freeze glycoprotein from polar fish (Guha et al. 2013). Of course, both mechanisms do not exclude each other. Antibodies to CD176/TF have been demonstrated to prevent TF-mediated metastatic spread (Shiogeoka et al. 1999) and to induce apoptosis (Yi et al. 2011). Furthermore, the expression of TF has been found to be correlated with invasive tumor growth (Limas and Lange 1986; Zebda et al. 1994), and interestingly also in a special case of normal cells (trophoblast cells) invading the decidua (Jeschke et al. 2002). The lectin Jacalin induces T lymphocyte activation following binding to TF on Jurkat cells (an acute T cell leukemia cell line, Baba et al. 2007).

An instructive example of how TF at a specific site can lead to a re-direction of differentiation is fibronectin (FN). Malignant FN (onfFN) differs from normal FN (norFN) by a glycosylation at the threonine of the sequence VTHPGY by either TF or its precursor, Tn, leading to a conformational change of the FN molecule which completely modifies its function (Matsuura et al. 1988). OnfFN, but not norFN, is able to induce EMT in carcinoma cells. Moreover, onfFN acts synergistically in this repect with the transforming growth factor, TGFβ1 (Ding et al. 2012). Interestingly, tumor MUC1 differs from normal epithelial MUC1 in a similar conformational change induced by *O*-glycosylation at the threonine of the sequence PDTRP with either TF or Tn (Karsten et al. 2005).

Taken together, direct and circumstantial evidence suggest that the TF disaccharide is typically found on proteins which are (cancer) stem cell markers or which are proteins with similar functions. Moreover, TF confers direct and indirect properties enhancing the malignancy of the cancer cell. Thereby TF is a characteristic example for the type of changes which occur on glycoprotein stem cell markers during malignant transformation and which, according to our hypothesis, make the difference between normal and cancer stem cell markers.

### Questions to be answered

The fact that the glycosylation of cellular glycoproteins is altered in cancer has been well known for decades (Hakomori 1989). Our hypothesis, however, does not simply extend this idea to stem cell markers but claims that this is not a random process. It appears to be selective with respect to the proteins as well as with respect to the glycans involved. This raises several questions, for instance, what is the reason for the apparent selectivity of expression of, e.g., CD176/TF (and probably certain other glycans) on stem cell marker molecules? We are at present unable to offer an explanation for this type of selectivity. However, remarkable selectivity of glycan changes has already been reported in other cases (Hernandez et al. 2007; Singh et al. 2001).

Furthermore, one may ask which other glycans from a great diversity of potential candidates (Hakomori 1989; Zhang et al. 1997) might be able to confer the property of being selectively expressed as CSC markers. Tumor specificity may be the most important qualifier. According to this, CD176/TF is a prime candidate. However, it remains open to what extent other known carbohydrate tumor markers such as, for instance, CD175 (Tn), CD175s (sialyl-Tn), CD174 (Lewis Y), CD15 (Lewis X), CD15s (sialyl-Lewis X), CA19-9 (sialyl-Lewis a), or some subtypes of A or H (blood group-related glycans) might also be carried on CSC marker proteins. So far only few data are available. Lewis Y is at present the second most likely CSC marker-specific glycan. It has been found co-expressed with CD44 in breast cancer tissues (Lin et al. 2010b). Tn expression apparently alternates with TF (Barrow et al. 2013), and has also been found on oncofetal fibronectin in exchange to TF (Matsuura et al. 1988). It may be that these different glycans indicate different stages of the malignant stem cell-progenitor-tumor end cell lineage. Lewis X is carried on CD147, a potential CSC marker (Miyauchi et al. 1990; Riethdorf et al. 2006), but also known as a normal stem cell marker (Hennen and Faissner 2012).

Our hypothesis applies so far essentially to stem cell markers which are mucin-like surface proteins, which predominantly carry *O*-glycans. *N*-glycans are also altered on cancer stem cells (Hemmoranta et al. 2007). Their suitability as CSC markers remains to be elucidated. However, strong support for our hypothesis comes from glycolipids, whose changes in malignant transformation and in EMT are well known (Hakomori 1996). Some of them are CSC markers (Table 2). For instance, both the globoside Gb3 and the ganglioside GD2 have been described as breast cancer stem cell markers (Gupta et al. 2012; Battula et al. 2012).

It should be mentioned that some stem cell markers are intracellular proteins, such as Oct-4 (Monk and Holding 2001) or nestin (Krupkova et al. 2010). Their glycosylation is different from that of surface proteins, and so are any deviations in cancer cells (Slawson and Hart 2011).

## Conclusions

The CSC concept, although well founded, has had to adapt to complex and partially adverse processes such as the role of EMT or the influence of the microenvironment on cancer stem cells (Medema 2013). The role of glycosylation of stem cells, and especially of stem cell markers, may add a further dimension to it.

If confirmed, this hypothesis has several consequences. First, stem cell markers which are found on normal as well as on tumor stem cells should be systematically analyzed for their glycan patterns in both circumstances. In particular, CSC markers should be examined for their potential expression of CD176/TF, CD175/Tn, and CD174/Lewis Y. Second, these tumor-related glycans could become very important or even crucial therapeutic targets. Third, targeting CD176/TF might also help to overcome the therapeutic problem of EMT, i.e. the generation of secondary cancer stem cells, because CD176/TF is expressed on oncofetal fibronectin, which plays a key role in this process (Matsuura et al. 1988).

In this connection the remarkably successful treatments of breast cancer patients by Georg F. Springer with a TF-carrying vaccine (Springer et al. 1994; Springer 1997) should be remembered. They may now be seen in a new light.
